# Oncogenic fusion transcript analysis identified ADAP1‐NOC4L, potentially associated with metastatic colorectal cancer

**DOI:** 10.1002/cam4.4943

**Published:** 2022-06-14

**Authors:** Amin Talebi, Soodabeh Shahidsales, Mohsen Aliakbarian, Masoud Pezeshki Rad, Mohammad Amin Kerachian

**Affiliations:** ^1^ Medical Genetics Research Center Mashhad University of Medical Sciences Mashhad Iran; ^2^ Faculty of Medicine, Department of Medical Genetics Mashhad University of Medical Sciences Mashhad Iran; ^3^ Cancer Research Center Mashhad University of Medical Sciences Mashhad Iran; ^4^ Faculty of Medicine, Surgical Oncology Research Center Mashhad University of Medical Sciences Mashhad Iran; ^5^ Faculty of Medicine, Department of Radiology Mashhad University of Medical Sciences Mashhad Iran; ^6^ Cancer Genetics Research Unit Reza Radiotherapy and Oncology Center Mashhad Iran

**Keywords:** functional study, fusion transcripts, metastatic colorectal cancer, RNA sequencing, transcript chimera

## Abstract

**Purpose:**

Fusion transcripts are transcriptome‐mediated alterations involved in tumorigenesis and are considered as diagnostic, prognostic, and therapeutic biomarkers. In metastatic colorectal carcinoma (mCRC), fusion transcripts are rarely reported. The main challenge is to identify driver chimeras with a significant role in cancer progression.

**Methods:**

In the present study, 86 RNA sequencing data samples were analyzed to discover driver fusion transcripts. Functional assays included clonogenic cell survival, wound‐healing, and transwell cell invasion. Quantitative expression analysis of epithelial‐mesenchymal transition (EMT), apoptotic regulators, and metastatic markers were examined for the candidate fusion genes. Kaplan–Meier survival analysis was performed using patient overall survival (OS).

**Results:**

A variety of driver fusions were identified. Fourteen fusion genes (51% of mCRC), each at least found in two mCRC samples, were determined as oncogenic fusion transcripts by in silico analysis of their functions. Among them, two recurrent chimeric transcripts confirmed by Sanger sequencing were selected. Positive expression of *ADAP1‐NOC4L* was significantly associated with an increased risk of poor OS in mCRC patients. In vitro transforming potential for the chimera, resulting from the fusion of *ADAP1* and *NOC4L* was assessed. Overexpression of this fusion gene increased cell proliferation and enhanced migration and invasion of CRC cells. In addition, it significantly upregulated EMT and anti‐apoptotic markers.

**Conclusions:**

*ADAP1‐NOC4L* transcript chimera, a driver chimera identified in this study, provides new insight into the underlying mechanisms involved in the development and spread of mCRC. It suggests the potential of RNA‐based alterations as novel targets for personalized medicine in clinical practice.

## Graphical abstract

ADAP1‐NOC4L transcript chimera, a new driver chimera identified in this study, provides new insight into the underlying mechanisms involved in the development and spread of metastasis. It suggests the potential of RNA‐based alterations as novel targets for personalized medicine in clinical practice.

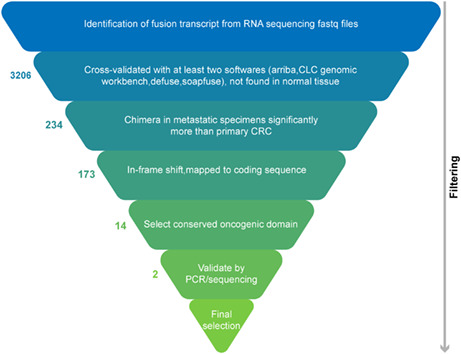

## 1 INTRODUCTION

Colorectal cancer (CRC) is one of the primary causes of cancer‐related morbidity and mortality around the world.^1^, ^2^ The metastatic spread of tumor cells to the liver, the most common target of tumor cell dissemination, occurs eventually in most patients with primary CRC, with median overall survival (OS) of 5–20 months, if not treated.^3^, ^4^

In the last two decades, with the introduction of novel therapeutic methods such as anti‐angiogenic and anti‐Epidermal Growth Factor Receptor (EGFR),^5^ the resectability rates of patients' tumors with liver metastases have impressively improved.^6^, ^7^ However, the problem that which patients with resectable disease and high‐risk features benefit from adjuvant therapy still remain unknown.^8^ As a result, predictive biomarkers of chemotherapeutic efficacy are needed to select the appropriate metastatic colorectal cancer (mCRC) treatment. They could lead to better selection of patients for treatment options, as well as predicting tumors with a higher aggressiveness and those resistant to treatments.^9^, ^10^, ^11^ In addition, the discovery of additional genetic events should provide new cancer‐related biomarkers important in early diagnosis, and prognosis. On the other hand, in order to generate more promising results in the decision‐making process of mCRC patients, a personalized approach regarding molecular profiling is required.^9^ As we move closer to precision medicine, new molecular abnormalities are discovered as drivers for tumor initiation and development, potentially revealing novel therapeutic targets.^12^ Recent advances over the last decade in tumor genomic testing have made it possible to extensively determine the mCRC molecular landscape.^13^

In this regard, fusion transcripts are transcriptome‐mediated rearrangements which had been shown to play a role in the development of several malignancies.^14^In many cases, the identification of fusion transcripts has diagnostic values (e.g., *FLI1*/*EWS* in Ewing Sarcoma) as determining a particular tumor subtype. They could also predict prognosis (e.g., the presence of gene fusions in embryonal rhabdomyosarcoma), or might be of therapeutic importance [e.g., *ALK* and *ROS1* in non‐small cell lung cancer (*NSCLC*)).^11^

In mCRC, several fusions including NTRK, ALK, ROS, RET, BRAF fusions with prognostic, predictive, or druggable targets potential have been defined, so far^15^, ^16^, ^17^, ^18^, ^19^However, a comprehensive picture of effective fusion transcripts in the pathogenesis and development of mCRC, is not well understood.

In research practice, discovering chimeras involves two main aims and approaches: (1) Identifying targetable fusions associated with known therapeutic agents regardless of tumor type and (2) detailed molecular examination of a tumor to discover specific tumor alterations that require appropriately targeted treatments.^20^ The critical issue in these two scenarios is the low frequency of discovered chimeras specially among solid tumors, which limits their pathogenetic and therapeutic relevance in trial studies and their applications in clinical settings.^21^ It should be noted that in some cases the presence of these fusions defines certain subtypes of the tumor that may benefit from specific treatments and should not be ignored. For example, RET rearrangements, in particular, characterize a subgroup of mCRC that is resistant to conventional anti‐EGFR treatments but may respond to RET inhibitors.^22^

Recurrent chimera appears to play a more significant role in disease pathogenesis; however, The critical aspect in these instances is that the driver chimeras must be distinguished from passengers, as they play essential roles in tumor development and progression.^11^ The biological function of the genes involved, as well as the in vitro and in vivo characterization of chimera functions, could be determined as discriminating factors between driver and passenger chimera.^14^ A more comprehensive range of malignancies could be analyzed by using public databases, allowing to discover more significant recurrent molecular changes.

The present study aims to investigate recurrent driver fusion transcripts at the transcriptome level to discover potentially important chimeras in mCRC pathogenesis by analyzing RNA sequencing raw data from multiple public datasets. Here, we assess the driving function which is relevance of candidate cases at an *in‐silico* level by evaluating functional domains in selected cases, followed by functional establishment in vitro on the candidate chimeras. In addition, the influence of candidate chimeras' expression on patient prognosis are also investigated.

## 2 METHODS

### 2.1 Data collection and samples

Paired‐end RNA sequencing fastq files were downloaded from *Sequence Read Archive (SRA)* database (https://www.ncbi.nlm.nih.gov/sra) of the National Center of Biotechnology Information (NCBI) with accession numbers: SRP060016, SRP095672, SRP029880, SRP078268, SRP222902. Eighty‐six RNA sequencing data obtained from 24 paired liver metastasis, primary tumors, and normal specimens as well as seven primary tumors with seven liver metastases were considered for initial analysis. In addition, 17 fresh tissues (11 liver metastasis, and 6 primary tumors) with 23 formalin‐fixed paraffin embedded (FFPE) tissues (12 liver metastasis and 11 primary tumors) from the archives of the Iranian biobank of Mashhad Sina and Imam Reza hospitals were included.

For FFPE tissues, briefly, sample areas with at least 90% tumor cells, without mucin‐rich glands or prominent inflammatory cellular infiltration were selected under microscopic examination on each glass slide and the matched area of paraffin‐embedded tissues were selected and re‐embedded. Then, up to four sections of FFPE tissues were cut, each with a thickness of up to 10 micrometers. Ethical approval for this study was given by the ethics committee of the Mashhad University of Medical Sciences, Mashhad, Iran (Approval ID: IR.MUMS.MEDICAL.REC.1399.106).

### 2.2 Chimeric transcript detection

**FIGURE 1:**
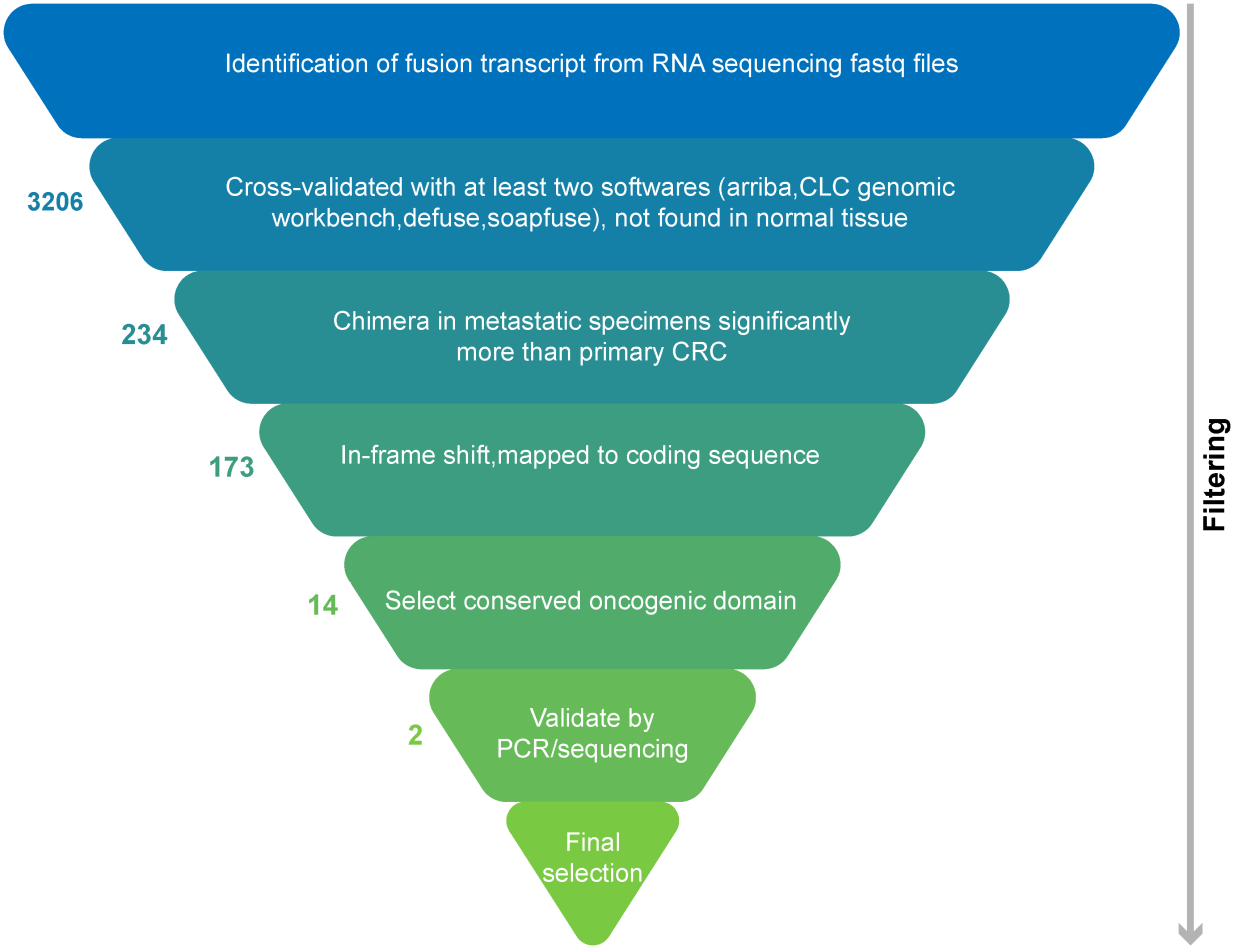
An overview representing the fusion transcript identification filtering pipeline from paired‐end RNA sequencing fastq data in metastatic tissue samples.

For fusion transcript detection, we used a combination of criteria's followed by a filtering pipeline (Figure 1). Four computational gene fusion detection tools were applied for fusion transcript investigation and cross‐validation, including Arriba,^23^ CLC genomic workbench 20 with a plugin1`6,^24^ SOAPfuse‐v1.27,^25^ and defuse‐0.6.2,^26^ which each has its own fusion detection algorithm and aligner. The pipelines were tuned to detect fusion transcripts in CLC genomic workbench 20 and SOAPfuse.

In order to find important potential protein‐coding fusion transcripts that are overexpressed in metastatic cells and potentially might be important in the development of metastatic clones, we used a filtering pipeline. The fusion transcripts that are significantly upregulated in metastatic specimens in comparison to primary CRC specimens, were included. Other inclusion criteria were the alignment of both fusion partners to the protein‐coding sequence and the fusion spanning reads with or without fusion crossing read, accepted greater than or equal to 5. Fusion transcripts that were out of the frame or also present in the normal colon tissue were excluded. In‐frame fusion transcripts were selected from the initial candidates, which were also determined by using AGFusion software^27^ or individually by checking each frame of the chimeric transcript separately.

We investigated the available literature and the fusion gene databases including TCGA,^28^ FusionGDB,^29^ ChimerDB 4.0,^30^ and Mitelman^31^ databases to recognize the novelty of the discovered fusions. Final candidate chimeras were evaluated to preserve driver domains by Oncofuse^32^ or a persistent open reading frame containing functional domains such as kinases. Human hg19/GRCH37 reference genome was considered for RNA‐Seq reads alignment.

### 2.3 Chimera validation

For fusion transcript validation in colon cancer cell lines and clinical tissue samples, quantitative reverse transcription polymerase chain reaction (RT‐qPCR) was carried out using gene specific primers spanning the fusion junction region, described in Table S1. The PCR products were analyzed on a 2% agarose gel and confirmed using Sanger sequencing with PCR amplification primers.

Total RNA was isolated using AccuZol™ Total RNA extraction kit (Bioneer Corporation, South Korea) from fresh tissues and SW48 CRC cell lines, purchased from Pasteur Institute of Iran. To extract RNA from FFPE tissues, RNeasy FFPE Kit (QIANGEN) was used. Subsequently, 1 μg of total RNA was reverse transcribed with AccuPower® RocketScript™ RT PreMix Kit (Bioneer Corporation).

PCR reactions were conducted for 5 min at 95°C, and 40 cycles for 30 s at 95°C, 30 s at melting temperature (TM) according to each primer set, 30 s at 72°C, and 10 min at 72°C. RT‐qPCR was performed with the same method to validate the transfection status of the transfected vectors into the CRC cell line. Furthermore, RT‐qPCR was performed for matrix metalloproteinase 9 (*MMP9*), N‐cadherin, fibronectin 1 (*FN1*), Vimentin, *Bcl2*, and *BAX* genes with specific primers designed for gene transcripts to evaluate the expression level of epithelial to mesenchymal transition (EMT) and metastatic as well as regulators of apoptosis biomarkers, respectively (Table S2). Quantitative RT‐PCR was performed by using the same cDNA referred above, and DNA was amplified using SYBRGreen, using LightCycler® 96 System (Roche, Germany). Relative expression levels of fusion transcripts were determined using the **2** ^−**ΔΔCt**^ method according to the MIQE guidelines.^33^ *GAPDH* gene expression was used as a reference gene for data normalization.

### 2.4 Construction of the ADAP1‐NOC4L encoding vector

Coding sequences of *ADAP1*‐*NOC4L* gene was used to construct vector for the chimera overexpression. After amplification in the cells containing this transcript using the forward primer 5′‐ATGGCCAAGGAGCGGC ‐3′ and reverse primer 5′‐ TCAGCTGAGCGTGAAGTGC ‐3′ from cDNAs isolated from SW48 cells, the sequence was cloned into pcDNA3.1/V5‐His B vector by one‐step ligation method.

### 2.5 Functional validation

For in vitro functional assessment of ADAP1‐NOC4L multiple assays including clonogenic cell survival assay, Wound‐healing assay, Transwell invasion assay, PI Annexin V apoptosis assay, and fusion expression analysis were performed. The detailed description of assays used are explained in file S1.

### 2.6 Chimera expression comparison analysis

The relative expression level of candidate chimera of sequentially validated positive samples in metastatic tissue compared to primary CRC were investigated by qPCR in 17 fresh tissues (11 liver metastasis and 6 primary tumors) and 23 formalin‐fixed paraffin‐embedded (FFPE) tissues (12 liver metastasis and 11 primary tumors).

### 2.7 Survival analysis for candidate genes

We examined the effect of the selected chimera expression levels on the OS rate of patients with primary and metastatic colorectal cancer.

### 2.8 Statistical analysis

Gene expression data are presented as the mean ± standard error of the mean. Data distribution was evaluated using the D'Agostino test. Comparison between two groups was conducted using the Student's *t* test and Mann–Whitney test. Kaplan–Meier curves and the log‐rank test were generated to assess the survival data. *p* value <0.05 was considered to indicate a statistically significant difference. Statistical analysis was performed using SPSS software version 22 (IBM Corp.)^34^ and GraphPad Prism software version 8 (GraphPad software, Inc.).^35^

## 3 RESULTS

### 3.1 Prediction of oncogenic chimeras in metastasis

Oncofuse software was applied to investigate preserved oncogenic domains in chimeras with the continuous open reading frame (ORF), which uses a Bayesian machine learning algorithm. Fusion transcripts with high driver scores that included exonic or coding regions were considered. In other cases, candidate driver fusion selection included cases in which at least one of the two fusion partners had a conserved oncogenic domain based on domain analysis with InterPro or Uniprot online tools, or the results of previous fusion transcript studies.

By using filtering pipeline criteria, we discovered a total of 3206 cross‐validated fusion transcripts with at least two out of four fusion detection tools. Among these fusion transcripts, 1617 were exclusively in metastatic tissues with 1245 mapped to the coding sequences, and 136 located in continuous ORFs (Table 1). Our results revealed that in some paired specimens (primary & metastasis CRC) the fusion was exclusively expressed in metastasis. Fourteen fusion transcripts (51% of mCRC) differentially upregulated in metastatic tissue were found to contain at least one preserved oncogenic domain or driver score >0.8, analyzed by Oncofuse (Table 2).

**TABLE 1:** Summary of final in‐frame driver fusion transcript discovered by filtering pipeline in mCRC

5′ Gene	5’ Gene_transcript_id	Breakpoint 1 Location	3’ Gene	3’ Gene_transcript_id	Breakpoint 2 Location	Strand	In‐frame	Functional domain	Type	Sample ID (type)	Read count
**EVI5**	ENST00000370331	chr1:93029199	**GFI1**	ENST00000427103	chr1:92944310	−/−	1 > 0	Zinc finger C2H2‐type	Intrachromosomal	SRR975592 (LM) SRR3827632 (LM) SRR10160732 (LM)	7 6 6
LNX1	ENST00000306888	chr4:54342920	FIP1L1	ENST00000358575	chr4:54324820	−/+	0 > 1	C3HC4 RING‐type	Intrachromosomal	SRR975587 (LM) SRR10160731 (LM)	5 5
PRKAA1	ENST00000397128	chr5:40771821	PTPN22	ENST00000528414	chr1:114372329	−/−	2 > 2	Serine/threonine‐protein kinase	Interchromosomal	SRR975589 (LM) SRR3827633 (LM)	5 5
MAP3K5	ENST00000359015	chr6:136901439	MAP7	ENST00000354570	chr6:136742937	−/−	2 > 2	Serine/threonine‐protein kinase	Intrachromosomal	SRR975587 (LM) SRR10160731 (LM)	6 5
**DCAF11**	ENST00000559115	chr14:24592286	**PSME1**	ENST00000382708	chr14:24606194	+/+	2 > 0	Proteasome activator pa28	Intrachromosomal	SRR975593 (LM) SRR975594 (LM) SRR10160731 (LM)	7 5 6
CLRN3	ENST00000368671	chr10:129690820	DOCK1	ENST00000280333	chr10:128768966	−/+	2 > 2	DNA‐binding domain	Intrachromosomal	SRR975587 (LM) SRR3827632 (LM)	8 5
THBS1	ENST00000260356	chr15:39885003	NFIB	ENST00000380959	chr9:14179779	+/−	0 > 2	Zinc finger, C2H2‐like	Interchromosomal	SRR10160731 (LM) SRR975588 (LM)	6 5
LIME1	ENST00000309546	chr20:62369852	SLC2A4RG	ENST00000266077	chr20:62371730	+/+	2 > 0	Zinc finger, C2H2‐like	Intrachromosomal	SRR975588 (LM) SRR975595 (LM)	9 6
LARS2	ENST00000265537	chr3:45565600	LIMD1	ENST00000273317	chr3:45677642	+/+	0 > 2	Zinc finger, LIM‐type	Intrachromosomal	SRR3827632 SRR3827633	5 5
**ADAP1**	ENST00000265846	chr7:959605	**NOC4L**	ENST00000330579	chr12:132635526	−/+	2 > 2	Arf GTPase activating protein /CCAATbinding factor	Interchromosomal	SRR5131510 (LM) SRR10160731 (LM) SRR987590 (LM) SRR975592 (LM) SRR975594 (LM) SRR10160732 (LM) SRR3827632 (LM)	6 8 6 5 7 6 6
SPPL2A	ENST00000261854	chr15:51012137	TRPM7	ENST00000560955	chr15:50955243	−/−	1 > 0	MHCK/EF2 kinase	Intrachromosomal	SRR975593 (LM) SRR10160731 (LM)	5 5
**APLF**	ENST00000303795	chr2:68753374	**SPTLC1**	ENST00000262554	chr9:94871116	+/−	2 > 0	SMAD/FHA domain	Interchromosomal	SRR4457128 (LM) SRR3827632 (LM) SRR10160732 (LM)	5 5 5
COL4A1	ENST00000375820	chr13:110817209	CAMK2D	ENST00000296402	chr4:114582928	−/−	2 > 2	calmodulin‐dependent protein kinase II	Interchromosomal	SRR975587 (LM) SRR975594 (LM)	6 5
**RNF43**	ENST00000407977	chr17:56494378	**SUPT4H1**	ENST00000225504	chr17:56428869	−/−	‐	Transcription elongation factor (*Spt4)*	Intrachromosomal	SRR975588 (LM) SRR987590 (LM) SRR975592 (LM) SRR975597 (LM) SRR4457128 (LM) SRR3827632 (LM) SRR975593 (LM) SRR10160731 (LM)	6 6 6 5 8 6 5 5

*Note*: Bold letters in 5'gene and 3'gene columns indicate finally selected chimeras. (−) and (+) in strand column shows sense and antisense strand and ordered from 5′ to 3′ gene.

Abbreviation: LM, liver metastasis.

**TABLE 2:** Detail representing types of fusion transcript regarding unique tissue type‐specific and also (*) Shows shared chimeras among liver metastasis and primary colorectal cancer

	Liver metastasis (%)	Primary CRC (%)	Common*(%)
Intrachromosomal Interchromosomal	614 (37.9) 1003 (62.1)	838 (56.1) 658 (43.9)	74 (79.5) 19 (20.5)
Coding Non‐coding	1245 (76.9) 372 (23.1)	1137 (76) 359 (24)	73 (78.5) 20 (21.5)
In‐frame Out of frame	136 (8.5) 1481 (91.5)	47(3.1) 1449 (96.9)	5 (5.3) 88 (94.7)

**FIGURE 2:**
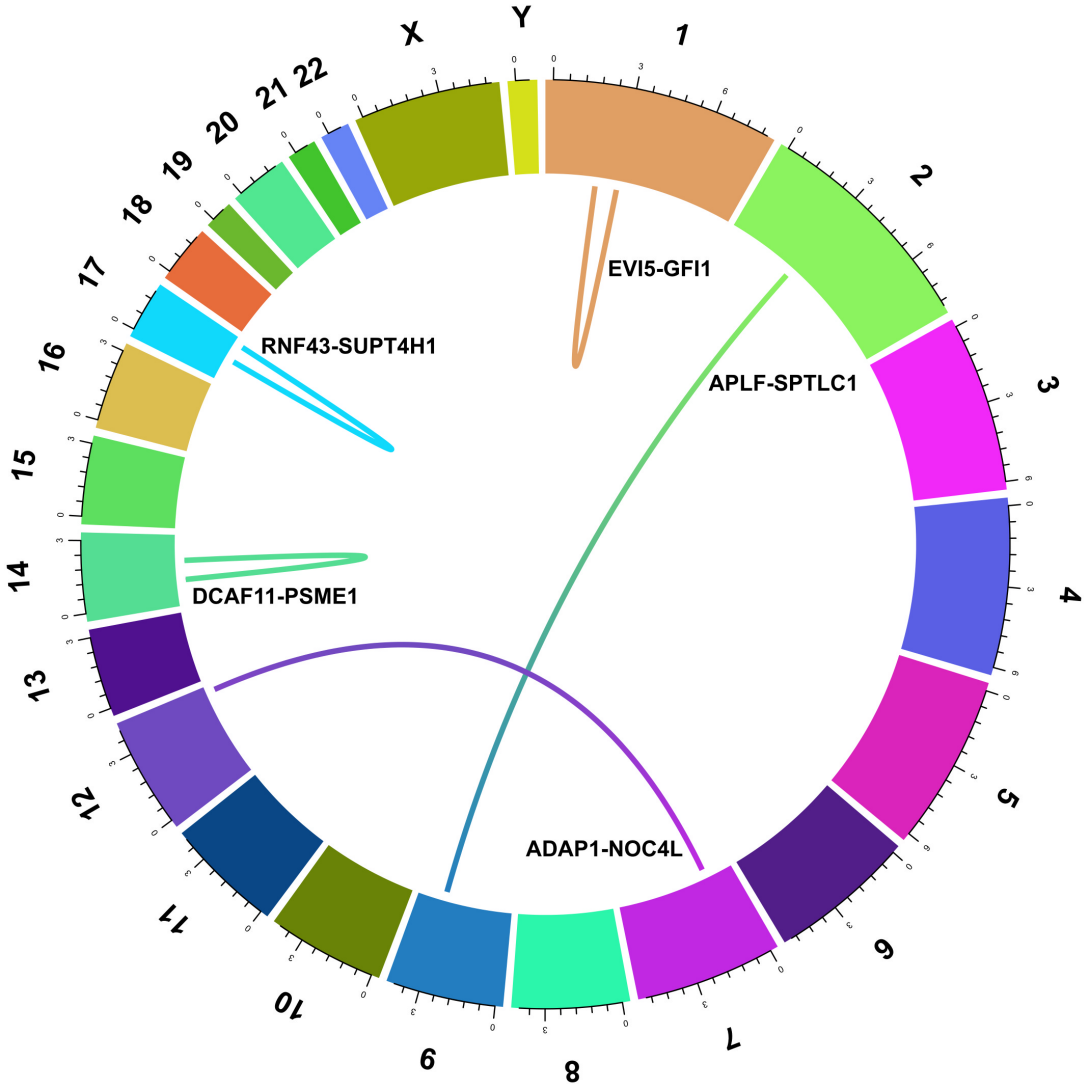
Circular view of the final 5 candidate chimeras with higher driver score or frequency representing the chromosomal regions involved, associated with each gene. ADAP1‐NOC4L and APLF‐SPTC1 fusions are illustrated with interchromosomal mechanisms and other fusions in this image are characterized by intrachromosomal mechanisms.

**FIGURE 3:**
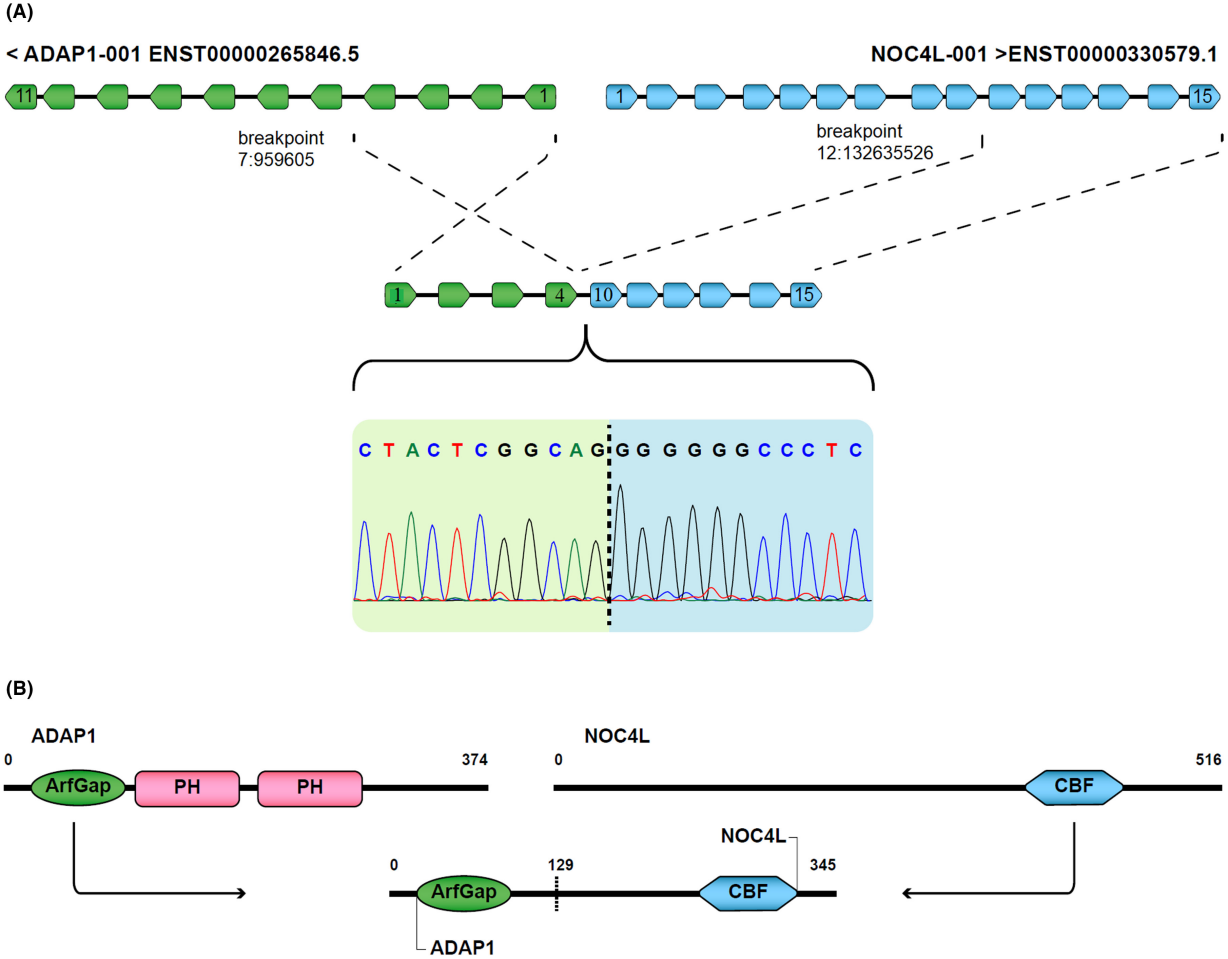
Schematic view of ADAP1‐NOC4L fusion and the domains A. ADAP1‐NOC4L fusion resulting from the binding of exon 4 of the ADAP1 gene and exon 10 of the NOC4L gene and electropherogram of the Sanger sequencing validation in the cell line expressing this fusion. B. Indicates the preservation of the ArfGap domain with GTPase function in the 5 ‘region and CBF domain in the 3’ end of the putative fusion protein.

**FIGURE 4:**
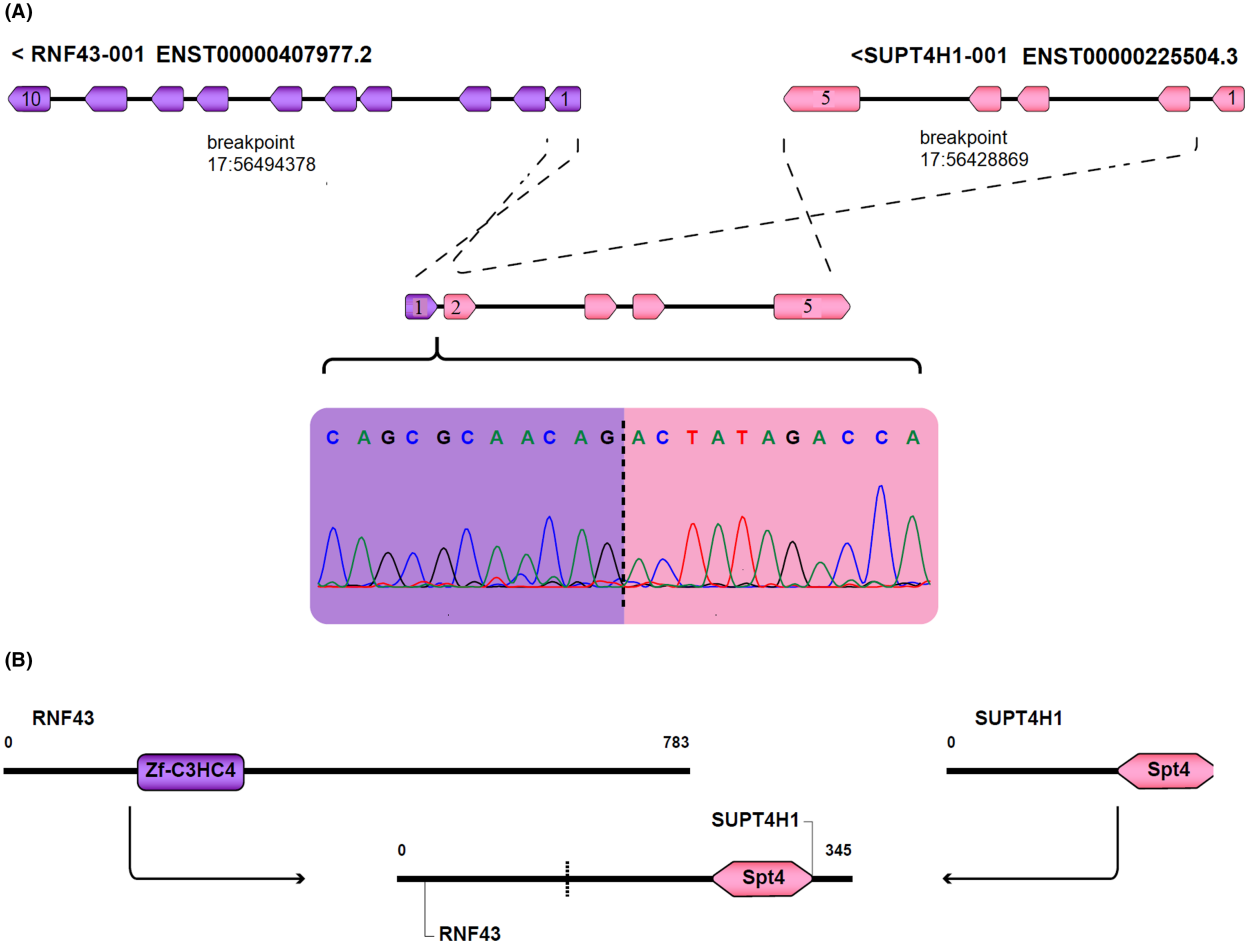
Schematic representation of RNF43‐SUPT4H1 fusion and its domains (A). The RNF43‐SUPT4H1 fusion is the result of the binding of the 5'UTR region of the RNF43 transcript to exon 2 of the SUPTH1 transcript, and the electropherogram shows Sanger sequencing validation in the expressing cell line. (B) Indicates the preservation of the SPT4 domain in the 3 ‘fusion region. This domain is involved in transcription regulation and is predicted to be involved in the oncogenic activity of this fusion.

Finally, five candidates fusion transcripts in 12 metastatic tissue samples (39% of mCRC samples) including two and previously known fusion transcripts *ADAP1‐NOC4l* found in seven (23%) mCRC specimens and also *RNF43‐SUPT4H1* discovered in eight (26%) of mCRC specimens and three chimeric transcript with highest driver score (>80%) found exclusively in metastatic samples were selected for validation with RT‐PCR and Sanger sequencing (Table 2, Figure 2). The two recurrent fusions (*ADAP1*‐*NOC4L* and *RNF43*‐*SUPTH1*) out of five chimeras were validated in SW48 and HT29 colon cancer cell lines as well as patient tissue samples (Figures 3, 4). These samples include 21 *ADAP1‐NOC4L* positive samples with 13(56%) mCRC specimens [7 (58%) FFPE and 6 (54%) fresh tissues], eight (47%) pCRC specimens [5 (45%) FFPE and 3 (50%) fresh tissues], 25 *RNF43‐SUPT4H1* positive samples with 15 (60%) mCRC specimens [8 (66%) FFPE and 7 (63%) fresh tissues] and 10 (40%) pCRC specimens [6 (54%) FFPE and 4 (66%) fresh tissues].

The *ADAP1*‐*NOC4L* fusion transcripts as a result of joining exon 4 *ADAP1* (ENST00000265846) to exon 10 *NOC4L* (ENST00000330579) had a continuous open reading frame (ORF) (Figure 3A). The DNA binding domains in *NOC4L* and the GTPase domain in *ADAP* were also preserved (Figure 3B). *RNF43*‐*SUPTH1* fusion transcript was identified as the result of joining the 5'UTR region of the *RNF43* (ENST00000407977) to exon 2 of the *SUPTH1* (ENST00000225504) (Figure 4A). Domain analysis with Uniport, and InterPro online database tools indicated an existence of a protected transcription elongation factor SPT4 domain in *SUPTH1* transcript (Figure 4B).

The other fusion transcripts identified in this study, have not been previously reported. They include *EVI5* (Ecotropic Viral Integration Site 5) ‐*GFI1* (Growth Factor Independent 1 Transcriptional Repressor) characterized by 5′ transcript Rab‐GTPase‐TBC domain and 3′ transcript Zinc finger C2H2 type preserved functional domain; *DCAF11* (DDB1 And CUL4 Associated Factor 11)‐*PSME1* (Proteasome Activator Subunit 1) contained 5'transcript WD domain repeats and 3'transcript proteasome activator pa28 alpha and beta subunit functional domain and *APLF* (Aprataxin And PNKP Like Factor)‐ *SPTLC1* (Serine Palmitoyltransferase Long Chain Base Subunit 1) marked with aminotransferase class I and II functional domain in 3′ region (Table 2, Figure S1).

### 3.2 Functional analysis of the ADAP1‐NOC4L chimera

For in vitro functional validation, the full coding sequence of *ADAP1*‐*NOC4L* (ENST00000265846: ENST00000330579 ADAP1‐001: NOC4L‐001) fusion transcript presented in SW48 cells was amplified. It was confirmed with RT‐PCR followed by Sanger sequencing, whereas the *ADAP1*‐*NOC4L* expression detected by RT‐PCR was significantly low in SW48 (Figure S2C) and absent in HT29 cell lines.

### 3.3 Clonogenic cell survival assay

**FIGURE 5:**
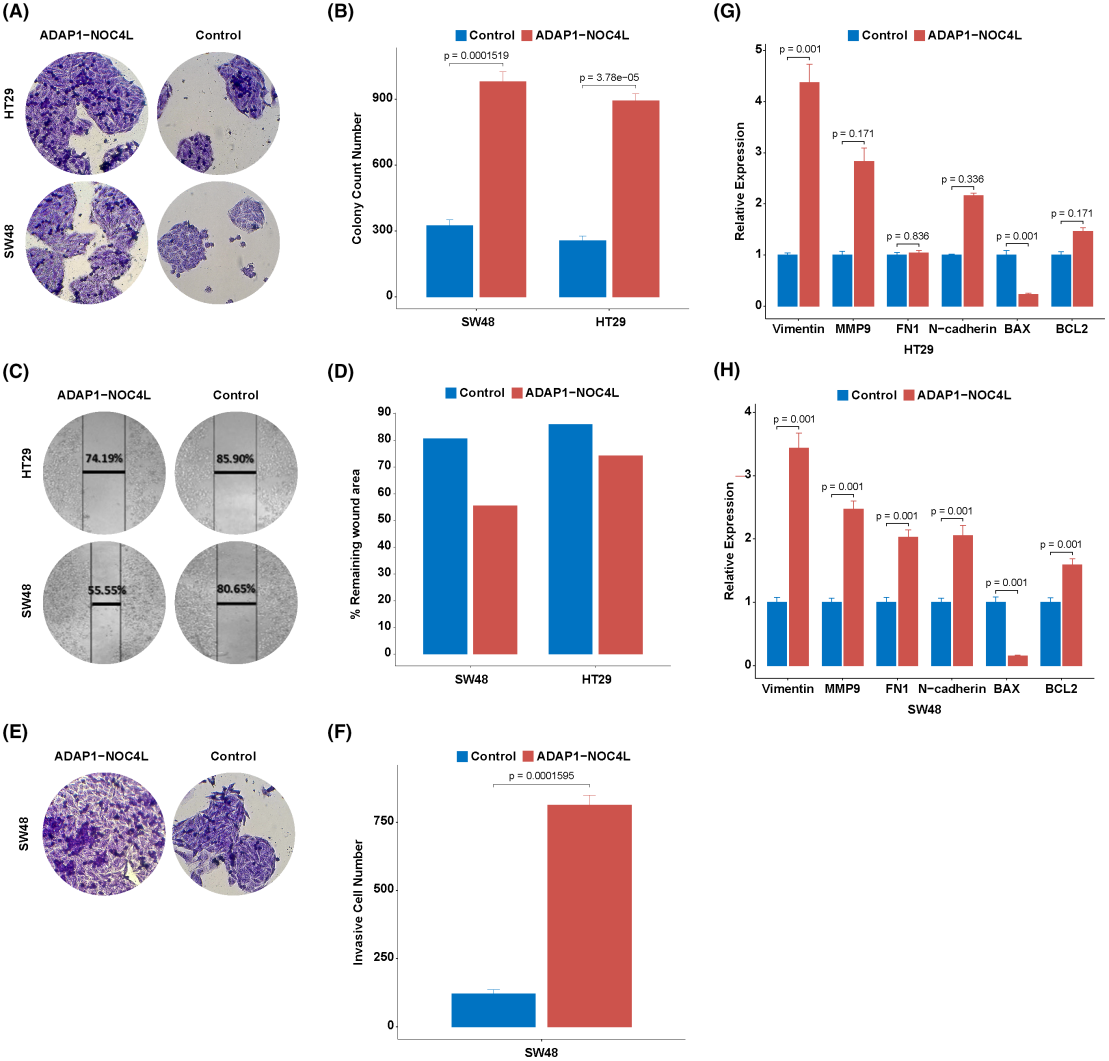
The effect of increasing ADAP1‐NOC4L over‐expression on functional and molecular characteristics of primary colon cancer cell lines. (A) and (B) Increased expression of the chimeric transcript has significantly increased the formation of cancer cell colonies (*p* < 0.001). C and D. shows 2D cell migration elevation in primary colon cancer. E and F. Increased expression of chimeric fusion caused a significant increase in cell invasion in SW48 cell line (*p* < 0.001). G. Increased ADAP1‐NOC4L expression significantly enhanced Vimentin expression (*p* = 0.001) and decreased BAX (apoptosis‐promoting biomarker) expression (*p* = 0.001) in HT29 cell line. H. Increased expression of ADAP1‐NOC4L significantly upregulated the expression of metastasis (EMT) and BCL2 markers (*p* = 0.001, *p* = 0.001) and decreased the expression of BAX (apoptosis‐promoting biomarker) in SW48 cell line (*p* = 0.001). EMT: Epithelial mesenchymal transition; MMP9: Matrix Metallopeptidase 9; FN1: Fibronectin 1.

A clonogenic assay was performed to determine the effect of the *ADAP1*‐*NOC4l* on the proliferation of cancer cell line SW48. The proliferation of over‐expressed SW48 and HT29 cell lines with *ADAP1*‐*NOC4L* increased at day 7 of transfection, compared to the negative control (*p* < 0.001) (Figure 5A,B).

### 3.4 Wound‐healing assay

The results of a wound‐healing assay revealed that the 2D migration of the SW48 and HT29 cells were increased following transfection with the overexpressing *ADAP1*‐*NOC4L* vector (Figure 5C,D).

### 3.5 Transwell cell invasion assay

To investigate the effects of fusion transcript on cancer progression, we conducted a transwell migration assay for SW48 cancer cell lines. Seventy‐two hours after transfection, SW48 cells harboring *ADAP1*‐*NOC4L* overexpression exhibited 6.5 fold increased invasiveness (*p* < 0.001) (Figure 5E,F).

### 3.6 PI Annexin V apoptosis assay

To further investigate the role of *ADAP1‐NOC4L* in the progression of CRC cells, we used the Annexin V propidium iodide staining. The percentage of apoptotic cells was determined by flow cytometric analysis. With the *ADAP1‐NOC4L* upregulation in SW48 cells, the population of early apoptotic cells (Annexin V‐positive, PI‐negative) decreased to 3.57% compared to negative control. The percentage of late apoptotic cells (positive for both Annexin V and PI) increased from 56.3% in negative control cells to 57.2% in SW48 cells upregulating *ADAP1‐NOC4L*.

The population of viable cells (Annexin V‐negative and PI‐negative) decreased to 13.2%, and there was an increase in the percentage of necrotic cells (Annexin V‐negative, PI‐positive), from 10.9% for the control group to 26% in the cells that overexpressed the fusion transcript (Figure S2A, B).

### 3.7 Expression analysis of metastasis biomarkers

The expression of MMP9 associated with metastasis was found by RT‐qPCR to be increased by 2.4 fold in SW48 (*p* = 0.001) and 2.8 fold in HT29 (*p* = 0.171) overexpressed cells. The results of qPCR also demonstrated higher expression of *FN1*, vimentin, and N‐cadherin in SW48 (*p* = 0.001, *p* = 0.001, *p* = 0.001) and HT29 cells containing fusion transcript (*p* = 0.836, *p* = 0.001, *p* = 0.336). *BCL2* anti‐apoptotic and *BAX* proapoptotic markers showed higher expression and downregulation in SW48 (*p* = 0.001, *p* = 0.001, *p* = 0.001) and HT29 (*p* = 0.001, *p* = 0.171) cells overexpressing *ADAP*1‐*NOC4L* chimeras compared to cells harboring empty vectors (mock), respectively (Figure 5G,H).

### 3.8 Chimera expression level comparsion

**FIGURE 6:**
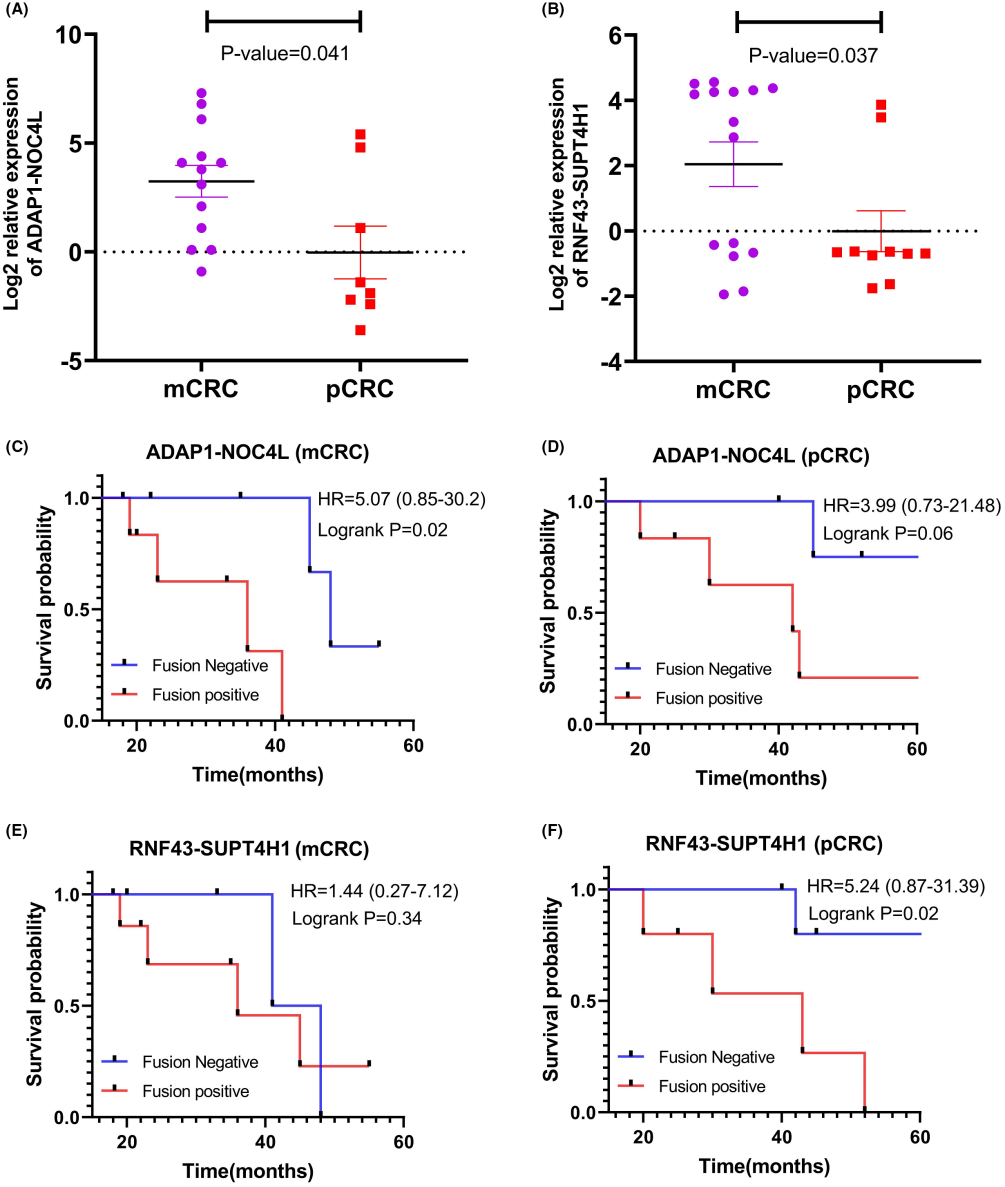
Relative expression comparison and overall survival analysis of chimeric transcripts candidates in fusion‐positive liver metastatic and primary CRC tumor. (A) Shows an increase in the relative expression of ADAP1‐NOC4L in metastatic colon cancer compared to the primary tumor (*p* = 0.041). (B) The relative expression of RNF43‐SUPT4H1 is also shown elevation in metastatic colon cancer compared to the primary tumor (*p* = 0.037). (C) Patients with positive expression of ADAP1‐NOC4L had a significantly shorter survival time compared with those with the absence of ADAP1‐NOC4L expression in mCRC. (*p* = 0.02) and (D) pCRC (*p* = 0.04). (E) Positive expression of RNF43‐SUPT4H1 in mCRC patients had no effect (*p* = 0.34) and (F) in pCRC patients had a direct effect on shorter overall survival rate (*p* = 0.02). mCRC: Metastatic colorectal cancer, pCRC: Primary colorectal cancer.

To further investigate the expression level difference of sequentially validated fusion transcripts between metastatic and primary CRC tissues, we analyzed the 21 *ADAP1‐NOC4L* positive samples [13(56%) mCRC and 8(47%) pCRC] and 25 *RNF43‐SUPT4H1* positive samples [15(60%) mCRC and 10(40%) pCRC], and found that higher relative expression of both candidate fusion transcripts (*p* = 0.041, *p* = 0.037) in liver metastasis samples compared to primary CRC tissues (Figure 6A,B).

### 3.9 Survival analysis

The association between chimera expression and OS time was analyzed using theKaplan–Meier method to determine the prognostic value of *ADAP1‐NOC4L* and *RNF43‐SUPT4H1* in patients with pCRC and mCRC. Only cases with survival information were included, which resulted in a total of 23. The results demonstrated that patients with positive expression of *ADAP1‐NOC4L* had a significantly shorter OS time compared with those with the absence of *ADAP1‐NOC4L* expression in mCRC (*p* = 0.02) (Figure 6C). Patients with pCRC, with positive expression of *RNF43‐SUPT4H1*, exhibited a significantly shorter OS time than those with negative chimera expression (*p* = 0.02. (Figure 6F). In other cases, the fusions identified did not demonstrate a significant correlation with OS of patients (Figure 6D,E).

## 4 DISCUSSION

In the present study, we aimed to identify important recurrent driver fusion transcripts involved in mCRC pathogenesis. We discovered 14 fusions exclusively in mCRC patients through RNA sequencing raw data analysis of four different public databases including 12 novel fusions that were not previously reported and two previously known recurrent fusion transcripts. Finally, we validated in vitro the driver function of *ADAP1‐NOC4L* fusion transcripts in CRC cell lines.

Multiple studies have discovered several fusion genes in mCRC, including NTRK, ALK, ROS, RET, BRAF, and FGFR2 fusions genes, harboring a prognostic and/or predictive value or representing potentially candidates of targeted therapy..^15^, ^16^, ^17^, ^18^, ^19^, ^36^ By applying fusion gene databases such as TCGA, FusionGDB, ChimerDB 4.0, Mitelman databases, our filtering strategy discovered novel fusion transcripts in mCRC.

In the current study based on the presence of putative oncogenic domains in fusion partners, we found 14 potential driver fusion transcripts in metastatic CRC by using Oncofuse, listed in Table 2. The expression of functional proteins resulting from the driver chimeric transcripts have been shown to promote cancer development and invasive migration.^37^, ^38^ Thus, we suggest that these cancer‐type specific transcriptomic alterations may influence metastatic cell growth, development, and differentiation of primary CRC cells. In addition, it could potentially be utilized to contribute to the diagnosis of colorectal metastatic cancer at the histological examination or early detection in plasma using circulating tumor RNA specimens.^39^, ^40^, ^41^ Furthermore, studies to focus on tissue specificity could leverage these fusion transcripts as diagnostic biomarkers to identify the tissue of origin of the metastatic lesions in cases of diagnostic ambiguity.

In the present study, we compared the expression of *ADAP1‐NOC4L* and *RNF43‐SUPT4H1* between mCRC and pCRC patients and we found that the expression of these fusions was not limited to metastatic tissue and was expressed in varying quantities in primary CRC tissue.

*Big Bang* model of tumor evolution suggests that tumor metastatic potential is specified ab initio during early stages of tumor development and the metastatic tumors are in fact as a result of few subclones from previous molecularly determined cells.^4^, ^7^ A recent study by Simeonov et al. has supported this idea.^42^ From this perspective, it can be expected that some driver alteration including fusion transcript formation involved in the development of metastatic clones, previously developed in primary CRC, might be upregulated. In this aspect, therapeutic management similar to primary tumor tissue could be also effective on metastatic tumors.

Morever, Kaplan–Meier survival analysis revealed that the presence of *ADAP1‐NOC4L* in mCRC patients and *RNF43‐SUPT4H1* fusion transcripts in pCRC patients were significantly associated with a shorter OS time. The findings indicate that *ADAP1‐NOC4L* and *RNF43‐SUPT4H1* expression may be a potential molecular marker for predicting the development and prognosis of patients with CRC. However, due to the limited number of samples examined, the current results should be interpreted with caution.

In this study, we validated two final candidate fusion transcripts by RT‐PCR followed by Sanger sequencing (Figure 3A, 4A). *ADAP1*‐*NOC4L* transcript involves exon 10 of the *ADAP1* gene linked with exon 9 of the *NOC4L*. *ADAP1*(Arf‐GAP with dual PH domain‐containing protein 1) is a protein containing two main domains including Arf‐GAP which is a putative zinc finger with GTPase activating proteins (GAPs) and a Pleckstrin homology (PH) domain (Figure 3B). The Arf‐GAP domain is critical in endocytic recycling and cytoskeleton remodeling. PH domain, plays a role in recruiting proteins to different membranes, thus targeting them to appropriate cellular compartments or enabling them to interact with other components of the signal transduction pathways.^43^, ^44^ However, the association between *ADAP1* and tumorigenesis has only been rarely investigated. By using high throughput approaches, numerous fusion transcripts, including *ADAP1* in the 3′ regions, have been described in different tumors.^30^, ^45^ Only a handful of them have been functionally proven, and there is only one known study on *ADAP1*‐*NOC4L*. In their research, Oga et al. introduced multiple ADAP1‐based in‐frame fusion transcripts. *ADAP1*‐*NOC4L* was reported out of frame, contrary to our study in which the ORF of the *ADAP1*‐*NOC4L* fusion was preserved. CBF is also critical in the function of *NOC4L*‐based fusion genes. As a result, we hypothesized that the excessive *ADAP1*‐*NOC4L* expression could contribute to the metastatic dissemination of a subset of primary colon cancer cells.

We further examined how *ADAP1*‐*NOC4L* is involved in CRC metastatic progression, not previously investigated. In order to validate the biological functions of the *ADAP1*‐*NOC4L* fusion transcript; first, we performed a functional analysis of *ADAP1*‐*NOC4L* fusion transcript in vitro. Overexpression of *ADAP1*‐*NOC4L* significantly increased cell growth and migration, compared to cells transfected with an empty vector. Then, we found that *ADAP1*‐*NOC4L* enhanced EMT in SW48 and HT29 cell lines. It has been shown that EMT is associated with invasion and metastasis in numerous carcinomas.^46^, ^47^, ^48^ In the last decade, a growing number of studies have demonstrated the critical involvement of EMT in the dissemination of various carcinomas including CRC.^49^, ^50^ Our results revealed that the mRNA level of EMT markers increased in SW48 and HT29 cells, suggesting that *ADAP1*‐*NOC4L* could promote EMT in CRC. It has been widely proposed that Bcl‐2 expression in cancer patient samples can promote cell migration, invasion, and metastasis by inducing MMP9 protein expression in various tumors.^51^ In contrast, cell invasion has been shown to be impeded by BAX and other cell death inducers and negative regulators of apoptosis such as BAK.^52^ In our study, *ADAP1*‐*NOC4L* overexpression increased cell motility and invasiveness, which was in the same way as *MMP9*, *BCL2* expression, and *BAX* downregulation and thus could be inferred to be effective in colon carcinoma cell metastasis.

*RNF43*‐*SUPT4H1* contains the joining of the 5'UTR region of *RNF43* RNA and exon 2 of *SUPT4H1* RNA which is a read‐through transcript and is likely not related to structural variation (Figure 4A). The RNF43‐SUPT4H1 predicted protein lacks all RNF43 domains but has preserved SPT4 domain of *SUPT4H1* (Figure 4B). It is likely to have nuclear localization and acts in a similar way to the wild‐type SUPT4H1 transcript. A study by Lee et al. showed that this fusion transcript commonly occurs in primary CRC samples.^37^ They discovered that *RNF43*‐*SUPT4H1* is prevalent in a variety of cell lines, including DLD‐1, HT29, HCT116, and HCT15. They also functionally validated the driver potential of this chimera in primary CRC cell lines; but it was not identified in metastatic tissues in an early bioinformatics analysis with SOAPfuse. However, in our study, bioinformatics analysis with CLC Genomics Workbench software showed differential expression metastatic samples, which was cross‐validated by Arriba and SOAPfuse. In addition, we elucidated the presence of this chimera in the SW48 cell line among different CRC cell lines (Figure 4A). In agreement with the previous similar study,^37^ we also speculated that this fusion event is a frequent molecular alteration and can serve as a potential diagnostic biomarker in metastatic CRC.

In the current study, we confirmed the expression of *ADAP1‐NOC4L* in mCRC patients' tissue samples and discovered discrepancies regarding the frequency of these fusion transcripts in mCRC between public data analysis and our patient clinical samples. In our specimens, *ADAP1‐NOC4L* fusion‐positive samples were identified in 56% of our mCRC tissues. In comparison, public data analysis revealed that only 23% of mCRC samples expressed fusion genes. These discrepancies have been also observed among other similar studies performed on fusion discovery in solid tumors^11^, ^21^, ^38^ and may be due to differences in sample collection, preparation, quality control, and bioinformatics analysis pipelines to select fusion transcripts.^53^

Here, we used several computational fusion detection tools such as Arriba 2.1.0,^23^ SOAPfuse‐v1.27,^25^ CLC Genomics Workbench 20,^24^ and defuse‐0.6.2^26^ to identify fusion events at the transcriptome level. Although RNA sequencing is a highly sensitive method for identifying RNA fusions, it is error prone at various stages such as pre‐sequencing and sequencing.^54^, ^55^ In addition, different fusion detection algorithms differ significantly in sensitivity and specificity, and therefore the use of different pipelines and experimental confirmation of results seems crucial to reduce false positive and negative results,^14^, ^53^, ^56^ which could be a major strength of the current study.

Another strength of this study is the investigation of recurrent fusions. As previously noted, fusion transcripts in solid tumors are uncommon and infrequently occur among individuals.^21^ Surprisingly, none of the fusion transcripts identified in Choi et al. study^38^ were detected in our analysis, even when using the identical (defuse) algorithms in metastatic or primary cancer specimens. In this regard, chimeras with higher frequency are clinically more important. The exploitation of public samples allows the possibility of studying a more comprehensive range of specimens. As a result, the possibility of discovering clinically significant genetic alterations rises.

There are some limitations in this study that could be addressed in future research. First, the limited number of samples available and second, the unavailability of patients' clinical data, which has restricted more comprehensive examination of the current discoveries. Another limitation is that the algorithms we used were unable to discriminate fusions happening at the transcriptome level from those occurring at the genome level. Future research is needed to gain complete knowledge of the selected fusions. Furthermore, additional investigations are recommended by using animal models of metastasis to investigate the role of fusion proteins in conferring the complex multistep process of metastasis as well as further wet lab research to assess the specific pathways involved in metastasis.

## 5 CONCLUSION

In the present study, we identified multiple novel chimeras as well as 2 recurrent driver protein‐coding fusion RNAs (*ADAP1‐NOC4L* and *RNF43‐SUPT4H1*) in mCRC. *In‐silico* analysis elucidated that the protein‐coding fusions account for a considerable amount of RNA‐based fusion in cancer. In vitro, functional evaluation of the *ADAP1‐NOC4L* chimeric transcript revealed a potential contributory effect in EMT and metastasizing the primary CRC. Survival analysis showed a prognostic biomarker potentiality of *ADAP1‐NOC4L* and *RNF43‐SUPT4H1* in metastatic and primary CRC, respectively.

## AUTHOR CONTRIBUTIONS

Mohammad Amin Kerachian and Soodabeh Shahidsales designed the experiments. Amin Talebi performed the experiments and analyzed the results. Mohsen Aliakbarian and Masoud Pezeshki Rad provided samples. All authors contributed to drafting the article and revising it.

## CONFLICT OF INTEREST

Authors have no financial conflicts of interests.

## ETHICS STATEMENT

Ethical approval for this study was given by the ethics committee of the Mashhad University of Medical Sciences, Mashhad, Iran (Approval ID: IR.MUMS.MEDICAL.REC.1399.106).

## AVAILABILITY OF DATA AND MATERIALS

The datasets generated and/or analyzed during the current study are available in the The Jackson Laboratory, Tumor Fusion Gene Data Portal [https://tumorfusions.org/], UTHealth hosts 2021 Commencement Ceremonies. fusion gene annotation DataBase [https://ccsm.uth.edu/FusionGDB/], ChimerDB 4.0 [http://www.kobic.re.kr/chimerdb/], Mitelman F JB, Mertens F Mitelman Database of Chromosome Aberrations and Gene Fusions in Cancer [https://mitelmandatabase.isb‐cgc.org/].

## INFORMED CONSENT

The patients/participants provided their written informed consent to participate in this study.

## Supporting information


Figure S1

Table S1

Appendix S1

## Data Availability

The datasets generated and/or analysed during the current study are available in the The Jackson Laboratory, Tumor Fusion Gene Data Portal [https://tumorfusions.org/], UTHealth hosts 2021 Commencement Ceremonies. fusion gene annotation DataBase [https://ccsm.uth.edu/FusionGDB/], ChimerDB 4.0 [http://www.kobic.re.kr/chimerdb/], Mitelman F JB, Mertens F Mitelman Database of Chromosome Aberrations and Gene Fusions in Cancer [https://mitelmandatabase.isb‐cgc.org/].
