# Supplementary material for: Oncogenic fusion transcript analysis identified ADAP1‐NOC4L, potentially associated with metastatic colorectal cancer
Source: Cancer Med. 2022 Jun 14;12(1):525–40. doi: 10.1002/cam4.4943 (PMC9844608; doi:10.1002/cam4.4943)
Supplement: Supplementary file 1 — Figure S1 [file CAM4-12-525-s001.docx]

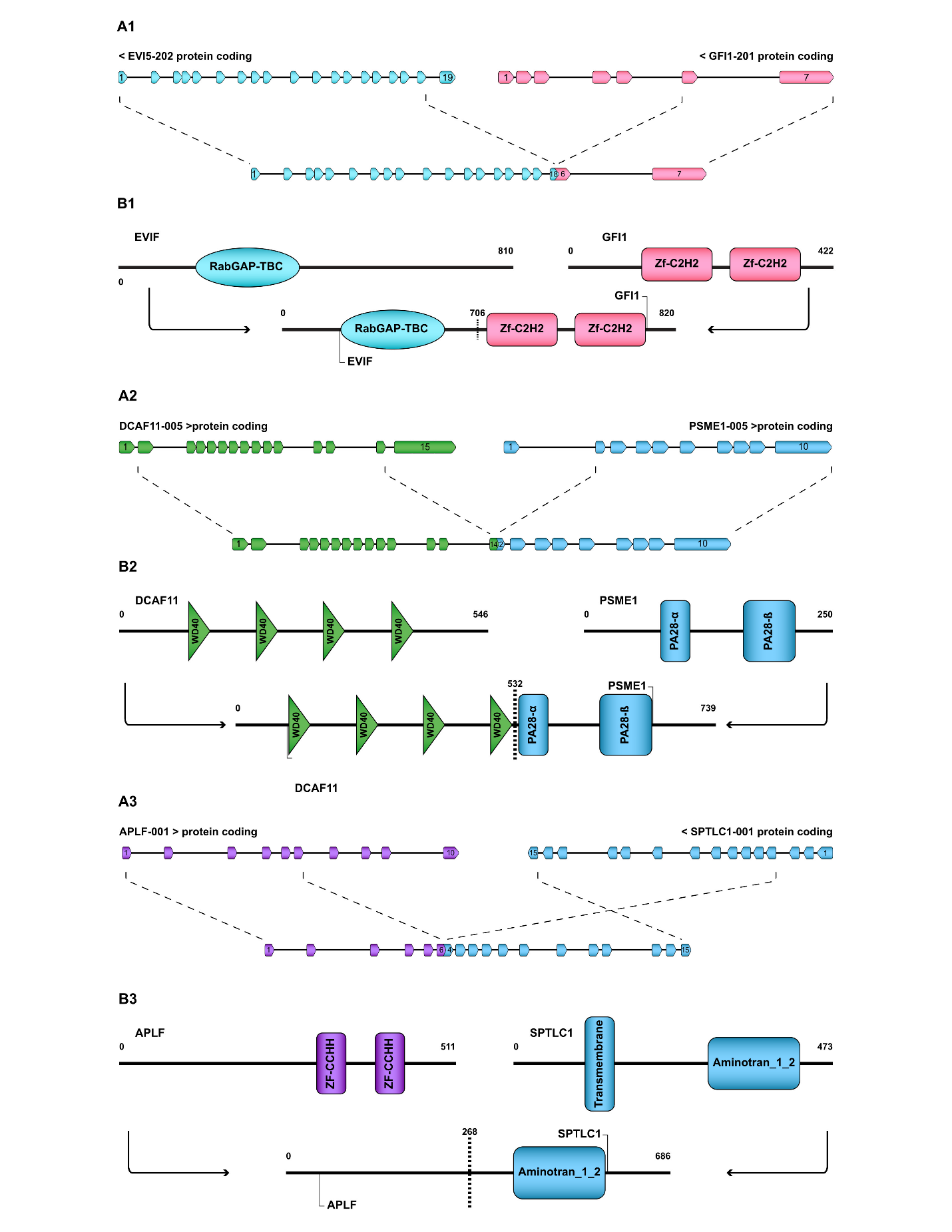


**Supplementary Fig. 1.** Fusion transcripts represented with their predicted functional domains. **A1**. Exon 18 of the donor EVI5 transcript and exon 6 of the acceptor GFI1 transcript are joined to form EVI5-GFI1. **B1**. The 5' transcript Rab-GTPase-TBC domain and the 3' transcript Zinc finger C2H2 type preserved functional domain are both present in the suggested protein. **A2**. DCAF11-PSME1 characterized by joining exon 14 of donor DCAF11 transcript and exon 2 of acceptor PSME1 transcript. **B2**. The potential protein has 5' transcript WD domain repeats and a functional 3' transcript proteasome activator pa28 alpha and beta subunit. **A3**. APLF-SPTLC1 characterized by joining exon 6 of donor APLF transcript and exon 3 of acceptor SPTLC1 transcript. **B3**. The hypothetical protein marked with aminotransferase class I and II functional domain in 3’ region.


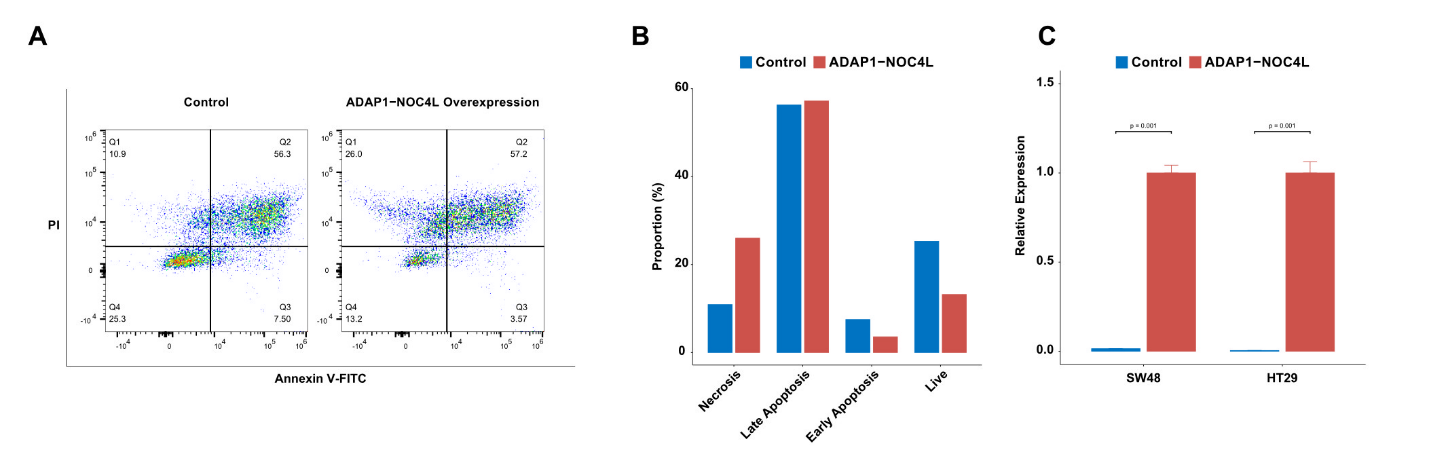


**Supplementary Fig. 2.** **A** Four-part plot derived from flow cytometric results of the PI Annexin V apoptosis assay in SW48 cells, showing higher expression of ADAP1-NOC4L relative to the control group. **B**. The rate of apoptosis in the SW48 stable transfected cell line against the negative control. In this cell line, ADAP1-NOC4L has a suppressive effect on the apoptotic rate. **C**. Comparison of ADAP1-NOC4L expression in two primary colon cancer cell lines, SW48 and HT29, before and after transfection with a vector encoding the fusion. Following transfection, SW48 expression increased significantly from a low baseline (p = 0.001). Fusion expression is detected after transfection in HT29 cells that do not express this fusion transcript in the basal state (p = 0.001).
