# Supplementary material for: Oncogenic fusion transcript analysis identified ADAP1‐NOC4L, potentially associated with metastatic colorectal cancer
Source: Cancer Med. 2022 Jun 14;12(1):525–40. doi: 10.1002/cam4.4943 (PMC9844608; doi:10.1002/cam4.4943)
Supplement: Supplementary file 2 — Table S1 [file CAM4-12-525-s003.docx]

| **Supplementary Table 1. Primer information for fusion gene validation** | | | |  | |
| --- | --- | --- | --- | --- | --- |
|  | **Primer sequence** | **Product size (bp)** | **Tm (℃)** | |  |
| *RNF43-SUPT4H1* | F: 5'-GAATTTGTTTCACCCCCG T-3' | 167 | 59.6 |  | |
|  | R: 5'-ATCGCAATGATTCCATCAAA-3' |  | 58.9 |  | |
| *ADAP1-NOC4L* | F: 5' - AGCCAGGTTTGAGTCCAAAG - 3' | 233 | 59.3 |  | |
|  | R: 5' - GAGCTTCCGGTAGAAGTCAGG - 3' |  | 60.4 |  | |
| *EVI5-GFI1* | F: 5' - CGGGAAGCAGATAGCATAGC - 3' | 240 | 60 |  | |
|  | R: 5' - TGGATAAGCAGGTGTGTGGA - 3' |  | 60.1 |  | |
| *DCAF11-PSME1* | F: 5' -CACTGGCAAAGTGGTTGTGT - 3' | 237 | 60 |  | |
|  | R: 5' - CATTGAGAGCTGGCTCCTTT - 3' |  | 59.9 |  | |
| *APLF-SPTLC1* | F: 5' - CAGCTAAACACAACCCAGCAAG - 3' | 249 | 60.2 |  | |
|  | R: 5' - CACAGTTTTGTGGCTTGGAG - 3' |  | 57.5 |  | |

| **Supplementary Table 2. Primer information for EMT and pro/anti apoptotic markers** | | | | |
| --- | --- | --- | --- | --- |
|  | **Primer sequence** | **Product size (bp)** | **Tm (℃)** |  |
| *fibronectin 1* | F: 5 '- AGCAGACCCAGCTTAGAGTT - 3' | 310 | 58.35 | |
|  | R: 5' - GCAGAAGTGTTTGGGTGACT - 3' |  | 58.32 | |
| *MMP9* | F: 5' - GCCACTACTGTGCCTTTGAGTC - 3' | 125 | 61.44 | |
|  | R: 5' - CCCTCAGAGAATCGCCAGTACT - 3' |  | 61.27 | |
| *Vimentin* | F: 5' - AGGCAAAGCAGGAGTCCACTGA - 3' | 100 | 63.88 | |
|  | R: 5' - ATCTGGCGTTCCAGGGACTCAT - 3' |  | 63.49 | |
| *N-cadherin* | F: 5' - CCTCCAGAGTTTACTGCCATGAC - 3' | 149 | 60.93 | |
|  | R: 5' - GTAGGATCTCCGCCACTGATTC - 3' |  | 60.29 | |
| *BCL2* | F: 5' -ATCGCCCTGTGGATGACTGAGT - 3' | 127 | 63.47 | |
|  | R: 5' - GCCAGGAGAAATCAAACAGAGGC- 3' |  | 61.98 | |
| *BAX* | F: 5' - TCAGGATGCGTCCACCAAGAAG - 3' | 103 | 62.57 | |
|  | R: 5' -TGTGTCCACGGCGGCAATCATC - 3' |  | 65.91 | |
