# Supplementary material for: Oncogenic fusion transcript analysis identified ADAP1‐NOC4L, potentially associated with metastatic colorectal cancer
Source: Cancer Med. 2022 Jun 14;12(1):525–40. doi: 10.1002/cam4.4943 (PMC9844608; doi:10.1002/cam4.4943)
Supplement: Supplementary file 3 — Appendix S1 [file CAM4-12-525-s002.pdf]

Functional validation assays:

Clonogenic cell survival assay

For measurement of colony formation, a stable SW48 cell line which was positive for wild-type ADAP1-NOC4L expression and HT29 cell line with no ADAP1-NOC4L basal expression transfected with the chimeric transcript vector or mock-transfected were seeded into six-well plates in a range of 7×10^0 to 1×10^4 cells and allowed to adhere. Following adherence, the cell culture medium was refreshed (2 ml/well in most experiments). Cells were subsequently incubated at 37 °C for 7 days, and cell growth of all six-well plates of a given cell line was determined simultaneously.

Fixation and staining were performed using 80% ethanol containing 0.5% w/v crystal violet (Sigma Aldrich, Taufkirchen, Germany). Colonies of ≥ 50 cells were counted under a stereomicroscope. Depending on cell morphology and colony size, counting was performed at 10- to 40-fold magnification.

Wound-healing assay

The stable SW48 and HT29 cell lines transfected with chimeric plasmid or mock (negative control) plasmid were seeded into 6-well plates in serum-free RPMI and DMEM respectively, 24 h before a wound was created. After the cells reached a confluency of 80%, a vertical linear scratch was created using a 10-µl pipette tip. The cells were subsequently washed with a culture medium without FBS. Cell migration across the wound was monitored under a microscope at 24 or 48 h. The width of the wound gap was measured using Image-Pro Plus (Media Cybernetics, Rockville, MD, USA). The ratio of the remaining wound area relative to the initial wound area was calculated as a wound closure percent.

Transwell invasion assay

Invasion property was examined by using a 24-well plate with 8-µm pore size inserts precoated with Matrigel (BD Biosciences). Cell suspension was seeded (1×10^5 cells/well) in the upper

chamber of a culture plate. A total of 800 μl of medium supplemented with 10% FBS was added to the lower chamber. After 72 h of incubation at 37℃, the non-invasive cells remaining in the upper chamber were removed using a cotton swab. The cells which passed through the inserts in the lower chamber were fixed with methanol and stained with 0.5% crystal violet at room temperature for 20 min. The invading cells were imaged using digital microscopy (Olympus, Japan). The number of invasive tumor cells was determined from five randomly selected 20× fields for each experiment and averaged.

PI Annexin V apoptosis assay

Apoptosis assay was performed using an Annexin-V-FITC apoptosis detection kit (BD, Franklin Lakes, NJ, USA). following the manufacturer's instruction. Briefly, cells were harvested using trypsin, washed twice with ice-cold PBS, and resuspended in 100 μl flow cytometry binding buffer. After the addition of 5 μl Annexin V-FITC and 5 μl propidium iodide, the cells were incubated in the dark for 15 min at room temperature. The apoptotic cells were analyzed by FACS Calibur flow cytometer (BD Biosciences, CA, USA).
